# Using Social Media to Target Cancer Prevention in Young Adults: Viewpoint

**DOI:** 10.2196/jmir.8882

**Published:** 2018-06-05

**Authors:** Urmimala Sarkar, Gem M Le, Courtney R Lyles, Danielle Ramo, Eleni Linos, Kirsten Bibbins-Domingo

**Affiliations:** ^1^ Center for Vulnerable Populations Division of General Internal Medicine University of California, San Francisco San Francisco, CA United States; ^2^ Weill Institute of Neurosciences Department of Psychiatry University of California, San Francisco San Francisco, CA United States; ^3^ Department of Dermatology School of Medicine University of California, San Francisco San Francisco, CA United States; ^4^ Department of Epidemiology and Biostatistics School of Medicine University of California, San Francisco San Francisco, CA United States

**Keywords:** cancer, prevention & control, young adult, behavior, social media

## Abstract

Focusing on primary cancer prevention can reduce its incidence. Changing health behaviors is critical to cancer prevention. Modifiable cancer risk factors include lifestyle behaviors related to vaccination, physical activity, weight control and maintenance, alcohol consumption, and tobacco use. These health habits are often formed in young adulthood, a life stage which currently intersects with the growing population of *digital natives* whose childhood occurred in the internet era. Social media is a critical communication medium to reach this population of digital natives. Using a life course perspective, the purpose of this viewpoint paper is to describe the current landscape of nascent research using social media to target cancer prevention efforts in young adults and propose future directions to strengthen the scientific knowledge supporting social media strategies to promote cancer prevention behaviors. Leveraging social media as a health promotion tool is a promising strategy to impact modifiable behavioral risk factors for cancer and warrants further research on developing effective communication strategies in young adults to prevent cancer in the future generations.

## Introduction

Cancer is a leading cause of death in the United States and a major growing public health burden. Primary prevention is an important strategy of focus as the burgeoning scientific research supports the notion that a large portion of cancer is preventable [1,2]. Although the etiology of cancer is multifactorial and complex and differs across specific types of cancer, it has been well established that approximately 50% to 60% of all cancers can be reduced with behavior change such as vaccination, physical activity, weight control and maintenance, reducing alcohol consumption, and smoking cessation [3,4]. Given this context, it is critical for public health efforts to prioritize the fostering of positive health behaviors to reduce the future burden of cancer.

Many of these health behaviors are considered modifiable risk factors, and to an extent, may be more susceptible to change and influence during critical age periods over one’s life course. Cancer prevention efforts have traditionally focused on older adults aged 40 years and over, who tend to be eligible for most cancer screenings and have more health awareness as they naturally experience more health issues with aging. However, much less attention has been paid to cancer prevention strategies targeted to younger age demographics, such as those aged 18-29 years, and, in particular, to strategies tailored through the use of new media. It is imperative to target young adults to promote cancer prevention behaviors before cancer develops. This younger age group is a critical developmental period that can set the stage for forming mindsets and worldviews that will ultimately shape future health habits and lifestyles [5,6]. Although cancer does not commonly occur in this age group, it is important to focus on prevention earlier in life, as cancer exposures are generally thought to occur earlier in life and contribute to cancers that are more commonly diagnosed among those 40 years and older (eg, lung, breast, colorectal, and prostate). Cancer prevention behaviors include these upstream behaviors, which can be modified earlier in life and directly relevant to young adults, as well as the more proximal action of completing recommended cancer screening, which is generally not relevant to young adults for the most common cancers (breast, prostate, and colorectal cancers).

The generation of young adults born from 1995 onwards are considered *digital natives* and defined as people “born or brought up during the age of digital technology and therefore familiar with computers and the internet from an early age” [7]. Young adults aged 18-29 years are the most frequent users of social media; in 2016, 86% of them used at least one social media site [8] and 92% engaged with 2 or more devices simultaneously including mobile phones, tablets, PC, and TV [9]. Social media must be considered as a public health strategy in young adults, simply because it is embedded in their everyday lives. To effectively reach them, health communication must occur where they are, engaging in online platforms, and must also be tailored using effective cancer prevention messaging uniquely suited for particular online platforms. For example, Twitter messages are limited to 280 characters and cancer prevention messaging to younger populations must take into design the linguistic and cultural factors in how to effectively communicate and engage young adults through Twitter.

In this viewpoint paper, we focus on social media and past use in primary cancer prevention in the general population and discuss how these studies can be applied to young adults to reduce the burden of cancer in the next generation of older adults. We reflect on the current state of the field and offer discussion on how previous research has implications for considering measurement and theoretical issues in future directions of research. Specifically, we provide an example of theoretical considerations from our current work (Lyson et al. Social media as a tool to promote health awareness: results from an online cervical cancer prevention study. Under review, submitted April 2018), describe various types of studies using social media for health communication with young adult digital natives with supporting examples, highlight methodological considerations in conducting studies in this field, and propose to integrate the life course perspective of cancer prevention with new forms of media, both of which overlap in the focus on young adults and lifestyle behavior change to present a unique opportunity for researchers to test effective cancer prevention strategies using social media.

## Theoretical Considerations

**Figure 1:**
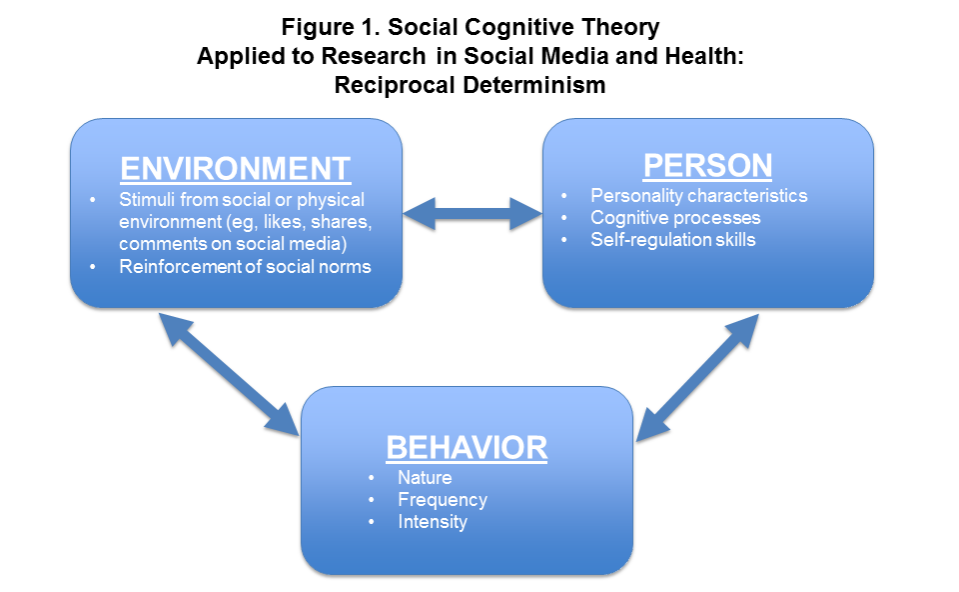
Reciprocal determinism in Bandura’s social cognitive theory for behavior change.

Theoretical considerations are an important component in conducting rigorous research in social media and health. Specifically, behavior change interventions are most effectively guided and tested by conceptual frameworks appropriate for the target audience. As an example, in our past and current work, Bandura’s social cognitive theory, an interpersonal-level health behavior theory [10], has been the most relevant theory to apply to research questions focused on social media influences on health behaviors. This theory encompasses social influences on health in a wide variety of settings and can naturally be extended to the social media environment. Social cognitive theory is used to explain how people learn behaviors by observing others and through vicarious reinforcement. It emphasizes reciprocal causation of behaviors between the self and society, in which personal factors in the form of cognitive, affective, and biological events, behavioral patterns, and environmental events all operate as interacting determinants that influence each other bidirectionally, that is, “reciprocal determinism” (Figure 1). As part of the environment, Web-based social media frames and reinforces social norms; social media sites have their own “rules” for reinforcement of messages and content in terms of likes, shares, and comments that are much more explicit than in everyday life.

When applied to social media communication, social cognitive theory suggests that new ideas, values, behavior patterns, and social practices are rapidly diffused worldwide through observational learning, in part through social networks. The concept of reciprocal determinism is critical to behavior change via Web-based social networks. Not only do individuals learn facts and information from social media but they are also actively shaping the social media sites to be broader networks for social change or political movements through their participation. This reciprocity sets the stage for peer-to-peer influence, as in studies in which groups interact via Web-based social media to address health issues. Furthermore, social media enriches the availability of public health data in the environment; in Bandura’s model, social media provides a “socially mediated pathway” to disseminate communication by linking people to social networks and community settings that provide natural incentives and continued personalized guidance for desired change. The social media activities of public health organizations, such as vaccination campaigns from the Centers for Disease Control (CDC) delivered via Twitter, allow for dissemination and reinforcement of health behaviors. The concept of “observational learning,” that individuals learn from watching others perform a given behavior, informs how behavior can spread via Web-based social media.

## Current Research in Social Media and Health

Public health research using social media takes place on a spectrum ranging from using social media as a real-time data source to engaging target populations online to influence health behaviors. Automated analysis of passively collected social media data can be used for disease or behavioral surveillance, including for early identification of disease outbreaks [11]. Public health organizations also deliver health information and health promotion messages using social media. In fact, the CDC has a social media toolkit intended to facilitate public health communication efforts via social media by partners and stakeholders [12]. This approach is unidirectional; experts deliver content to lay participants. The assumption in this approach is that populations at risk are willing and able to engage with health-related content and subsequently modify behavior. More recently, public health researchers have used social media to deliver health interventions that harness the immediacy of Web-based communication as well as the influence of Web-based social networks [13]. In social media intervention research, researchers interact with participants online, and participants may interact with each other. To augment our viewpoint discussion, we highlight various study designs that have been employed using social media data sources, provide supporting examples from the literature, and discuss implications for future research in social media and health.

### Observational Studies Using Social Media Data Sources

Because individuals, especially young adults, publicly share health information online, social media data can provide a robust data source for behaviors that are difficult to characterize and health data that are unavailable through traditional surveillance methods. This method of “mining” social media data for public health purposes is perhaps the most widely developed type of social media health research [14]. This type of observational research may be less prone to bias as people on social media typically do not act as though they are being observed for the purpose of research, in contrast to traditional research methods that explicitly recruit people to participate in research in academic settings and ask people to report on health-related behaviors. Myriad examples of this type of work exist across disparate public health domains including substance use [15], body weight-associated stigma [16], and infectious disease surveillance [11,17]. For example, Lyles et al performed this observational type of analysis for cervical cancer prevention discussions among young women on Twitter [18]. The analysis demonstrated that women do share publicly their experiences with cervical cancer screening, often with language encouraging peers to undergo screening as well. These user-generated health promotion messages are useful for characterizing public sentiment and informing public health messaging content. More recently, we analyzed Instagram data to characterize misuse of codeine on social media and found that codeine misuse was commonly represented with the ingestion of alcohol, cannabis, and/or benzodiazepines [19]. Our findings suggested that codeine misuse was represented as normalized behavior and found in mainstream commercialization of music and cartoons on social media. Because health behaviors are often difficult to capture in traditional observational research studies that rely on self-reported survey data, social media provides a unique lens through which stigmatized behaviors can be observed through a “fly on the wall” perspective.

This literature demonstrates that public health professionals can learn about community perceptions of cancer prevention-relevant behaviors by examining social media content. There is still much unrealized potential for connecting social media content and sentiment to real-world health behaviors. Thus far, one effective use of social media data has been in the area of “infoveillance” such as in influenza forecasting [20] and real-time outbreak identification [21]. Using geocode tags from social media data content could likewise be used to geographically pinpoint challenges and opportunities in cancer prevention behaviors; this methodology has been previously applied to infectious disease outbreak research. However, as a typical methodological concern for all self-reported data, the information on the user’s location is largely based on what is provided in their user profile, which may not be complete or accurate information. For Twitter data, it is estimated that about 1% to 2% of tweets are shown to be geotagged [22].

Observational studies using social media data have the advantage of accessing vast amounts of public data readily available in real time. This immediacy is a major advantage of using social media data to inform public health surveillance. However, methodological challenges remain in conducting rigorous and unbiased studies using social media data. Social media data are user-driven data and depend on the population who chooses to publicly share information. This is a self-selected group and may not represent the general population. Access to the internet and privacy concerns influence the likelihood of posting information online [23]. Internet access, particularly on mobile devices, is growing rapidly among young adults, and mobile internet is well suited to social media use. Privacy concerns are common, but younger adults compared with older adults are more likely to have shared personal information online [23], potentially enhancing generalizability in this age group. A second limitation of social media content as a public health data source is its unstructured nature, making comparisons across platforms or even individual messages challenging. Moreover, it is often impossible to verify the identity or other relevant details about individuals who post online. In general, social media posts often lack identification, demographic information, and other details. Social media data analysis must be interpreted in light of these inherent limitations.

### Unidirectional Mass Communication Health Promotion via Web-Based Social Media

Governmental organizations such as the CDC and the National Cancer Institute have used social media marketing strategies to deliver a wide array of health promotion content through multiple dissemination channels and platforms, such as blogs, Twitter, and Facebook [24]. Researchers have also used an online marketing approach for cancer prevention. As an example, Cidre-Serrano et al used Google AdWords to display skin cancer prevention messages on individuals’ search results page when users searched for tanning beds [25]. These prevention advertisements were displayed over 200,000 times over 2 months with a click-through ratio of 1%, which is generally considered sufficient for commercial purposes. Google for NonProfits and other Web-based platforms provide a limited amount of free advertising for nonprofit organizations, making this a low-cost approach for qualifying organizations. In general, the unidirectional strategy of “pushing” content at individuals has the advantages of being low-cost with a significant reach, as well as the ability to target content to specific high-risk groups (eg, young women who use tanning beds). However, data are lacking about the effect of health promotion messages delivered online. An example of planned work to address this gap would be to learn whether a Facebook advertising-based intervention aimed at reducing indoor tanning would shift knowledge and attitudes about indoor tanning and reduce individual intent to use tanning beds to ultimately prevent melanoma in high-risk groups.

Web-based social media is a powerful advertising and marketing tool as 88% of businesses use social media [26]; however, commercial entities have been shown to use social media to promote unhealthy behaviors. For example, Ricklefs et al documented the indoor tanning industry’s use of social media as a strategy for maintaining relationships with customers and to offer pricing deals that promote high-frequency tanning [27]. Similarly, e-cigarette advertising is prevalent on Twitter, particularly in states that limit other forms of tobacco advertising [28].

Provision of public health information or promotion via social media is subject to many of the same limitations as mass-media public health campaigns [29]. Social media messages are well integrated into the lives of users and can be easily accessible when they need it the most. The potential for health campaigns to go “viral,” increasing the audience size and impact, is a theoretical advantage of social media campaigns compared with traditional approaches, but it cannot be predicted or planned. Insomuch as content is easily accessible, it is, however, also easy to turn off. As with billboards, it remains unclear whether health content is reaching its intended audience. On social media, for example, many public health and medical professionals follow CDC on Twitter, but the extent of dissemination to the lay public is unknown. As with all unidirectional public health messaging, it is challenging to accurately assess the effect of such campaigns on health outcomes amidst the many other health influences in the individual’s environment. To measure the effectiveness of public health messages, innovative sampling and methods and proxy outcomes may be needed.

### Web-Based Social Media Interventions for Cancer Prevention Behaviors

Social media can also be used as a delivery platform for conducting intervention studies aimed at promoting health and wellness [13,30]. Web-based interventions have significant advantages: cost, ease of participation, and ability to scale up. These interventions can also harness the interaction dynamics of Web-based social networks and create positive peer-to-peer momentum for behavior change. For instance, in the area of smoking cessation, the Tobacco Status Project (TSP) is a Facebook intervention for young adult smokers combining messaging, peer-to-peer interaction, online counseling sessions, and group cognitive-behavioral sessions. A feasibility trial achieved 72% follow-up rates and an 18% rate of reported 7-day abstinence at 12 months (9% verified) [31]. Importantly, engagement in the intervention was high, with 92% participation in the full 3-month intervention [32]. These results demonstrate that Web-based social media platforms can be used to deliver behavioral interventions; however, the content, mode of delivery, and network structure all require careful planning and evaluation [33,34]. A clinical trial testing the efficacy of TSP on biochemically verified smoking abstinence is underway [35]. We believe that conducting interventions via social media platforms requires further study to understand the specific components that contribute to intervention effectiveness, such as the ideal intensity/timing/duration of the intervention, how/which Web-based social networks to access (general social networking vs disease-specific sites), the mix of peer-to-peer versus expert support for behavior change, how to escalate to “real-life” interventions such as pharmacologic treatment for tobacco (eg, nicotine replacement), and how to address Web-based misinformation and foster trust of information.

One of the major challenges in social media research is the rapid pace at which social media platforms evolve online and gain and lose popularity for certain segments of society. For example, Facebook has gained more users in the older age groups and has lost favor with younger age groups who have migrated to other platforms such as Snapchat. Research involving specific social media platforms can quickly become outdated as it can take several years for research studies to be funded, implemented, and ultimately published. This can be a frustrating challenge for researchers engaged in social media and health studies; although there are no easy solutions to this, there are possibilities to reframe the research questions to be more platform-agnostic and thus more widely applicable to the understanding how social media affects health behaviors. A more conceptual approach, driven by conceptual frameworks, to the research question can shed insights on constructs underlying social interactions that influence health behavior, as opposed to relying on specific platform. In considering the choice of platforms, researchers should prepare to be nimble and course-correct if they realize that the target audience or research question does not match the intended platform. Funded research should consider alternate platforms as part their research strategy and anticipate potential problems and alternative solutions to meet the needs of the research question.

Although social media interventions have the significant advantage of reaching people where they are, more complex health behaviors such as quitting smoking may require more intensive interventions beyond online social interactions. For instance, replacing in-person tobacco cessation counseling with online counseling allows participants to receive content without consuming transportation time, and at their convenience; however, there is a concern that delivering interventions online may dilute their effectiveness, especially because of the lack of personal connection. Moreover, many evidence-based interventions developed to be delivered in-person or via telephone require significant adaptation for Web-based social media [33], and reach, efficacy, and implementation may differ significantly. Future studies should incorporate rigorous methodological approaches in the design and evaluation of social media interventions by drawing on appropriate conceptual frameworks and evaluation methods from implementation sciences [36] regardless of whether they are newly developed or are adopted from existing interventions, because the “rules of engagement” online are so different from traditional health intervention environments.

Measuring outcomes is a methodological challenge in all types of studies. For social media and health research, there are various ways in which outcome measures can be obtained: (1) enrolling participants online and obtaining informed consent to follow participants for behavior change, (2) partnering with platforms to examine online actions (social media analytics such as click-throughs, page-viewing behaviors, purchases, etc), and (3) partnering with health systems for data linkage and online/clinic-hybrid interventions (linkage with electronic health records). These approaches combine traditional research methods of data collection (ie, direct data collection from participants through surveys) with innovative partnerships with social media platforms and health systems to provide a more comprehensive collection of outcome data to ascertain intervention effectiveness.

## Conclusion

In this new era of communication, social media has tremendous potential to improve public health as it has permeated society across all socioeconomic strata and races/ethnicities [37]. Young adults comprise a diverse population on social media, which has implications for addressing future disparities in cancer. The range of research described in this viewpoint paper harnesses a variety of disciplines, ranging from data science to social science. There is a need to ensure that multidisciplinary research teams have the appropriate expertise to conduct the research; the team’s composition should be driven by the expertise needed for the proposed research questions (data science, disease-specific/clinical expertise, behavioral science, communication sciences, public health professionals, social marketing experts, and qualitative and quantitative methods). Furthermore, research is needed to understand the effects as well as risks of using social media for cancer prevention in young adults to determine the impact on reducing the future burden of cancer. Use of social media as a health promotion tool seems most relevant to modifiable behavioral risk factors in young adults and warrants further research to prevent cancer in the next generation.

## Abbreviations

CDC: Centers for Disease Control
TSP: Tobacco Status Project
